# Improving Knowledge and Early Detection of Atrial Fibrillation through a Community-Based Opportunistic Screening Program: What’s Your Beat?

**DOI:** 10.3390/ijerph19116860

**Published:** 2022-06-03

**Authors:** Abubakar Ibrahim Jatau, Luke R. Bereznicki, Barbara C. Wimmer, Woldesellassie M. Bezabhe, Gregory M. Peterson

**Affiliations:** School of Pharmacy and Pharmacology, College of Health and Medicine, University of Tasmania, Hobart, TAS 7005, Australia; luke.bereznicki@utas.edu.au (L.R.B.); barbara.wimmer@utas.edu.au (B.C.W.); woldesellassie.bezabhe@utas.edu.au (W.M.B.); g.peterson@utas.edu.au (G.M.P.)

**Keywords:** atrial fibrillation, detection, knowledge, screening, stroke, elderly, community

## Abstract

A community-based opportunistic screening program was implemented to (i) improve atrial fibrillation (AF) awareness and detection and (ii) assess the performance of the Microlife WatchBP Home A for detecting AF when used in community screening. Screening sessions were conducted among people aged ≥ 65 years with no history of AF at public events across Tasmania, Australia. Participants with positive screening results were referred to their general medical practitioner for assessment. The device’s performance was assessed using the positive predictive value. A total of 1704 eligible participants were screened at 79 sessions. Of these people, 50 (2.9%) had a positive screening result. The device correctly identified AF in 22 (46.8%) participants with positive results. Among those with subsequently confirmed AF, 6 (27.3%) had a history of AF but were not aware of the diagnosis, and 16 (72.7%) were identified to have previously undiagnosed AF, with an overall prevalence of 0.9% (95% CI, 0.58 to 1.52). Oral anticoagulation therapy was initiated in 12 (87.5%) eligible participants. The positive predictive value of the device was 46.8% (95% CI, 33.3 to 60.7). Given the relatively low performance of the device, its application in community-based opportunistic screening programs for AF is unlikely to be cost-effective.

## 1. Introduction

Atrial fibrillation (AF) is a cardiovascular disorder affecting about 0.5% of the total world population [1], with an estimated five million new cases diagnosed every year [1]. People with AF have a five-fold increased risk of ischaemic stroke, a three-fold increased risk of heart failure, and a 40% to 90% increased risk of mortality compared to those without AF [2,3]. Evidence has shown that oral anticoagulant (OAC) therapy reduces stroke risk by 64% and mortality by 25% in those with diagnosed AF, compared with non-treatment [4]. AF can be asymptomatic in about one-third of people with AF [5]. These individuals are more likely to remain undiagnosed and experience an ischaemic stroke than those with symptomatic AF [6]. As shown in the Adelaide Stroke Registry data, those with previously undiagnosed AF may account for about 30% of all AF-related strokes [7]. Thus, there is a need for interventions to identify people with previously undiagnosed AF in the community.

Screening for AF has been recognised as one of the fundamental strategies for increasing AF detection and potentially reducing AF-related stroke, mortality, and healthcare costs [3]. Specifically, opportunistic screening was identified as a critical research priority towards improving AF diagnosis, management, and prognosis [3]. Opportunistic screening for AF by pulse palpitation or 12-lead electrocardiogram (ECG) rhythm strip has been recommended among people aged ≥ 65 years by European and Australian guidelines [3,8]. The Australian AF guideline further recommends opportunistic screening for AF in the community [8]. However, differences exist in the literature regarding the clinical benefits and cost-effectiveness of such screening [9,10]. For example, the United States AF guideline [11] and the UK National Screening review [10] do not recommend AF screening for asymptomatic individuals. Many experts have suggested that opportunistic screening could be considered for implementation based on the available evidence while awaiting results from on-going large randomised clinical trials (RCTs) [12].

Evidence has shown that community-based opportunistic screening can detect 0.5% to 3.0% of people as having previously undiagnosed AF [13] and could provide opportunities for delivering AF awareness campaigns in the community, particularly to those at risk of developing AF. Thus, the AF Screen International Collaboration and the National Screening Framework reports have recommended incorporating educational campaigns in screening programs to enhance AF awareness in the community [12,14]. However, only a few studies in the literature to date have incorporated AF awareness campaigns in screening programs.

The advent of new technology has simplified and made opportunistic screening for AF feasible in the community. A recent RCT demonstrated that new technologies increased the detection of new cases of AF 10-fold compared to routine practice [15]. An automated blood pressure (BP) monitor with an algorithm to detect AF (Microlife WatchBP Home-A, Microlife AG Swiss Corporation, Widnau, Switzerland) [16] has been recommended for opportunistic AF screening in primary healthcare by the UK NICE [17]. The diagnostic performance of Microlife WatchBP Home A has been validated in opportunistic screening within clinical settings among high-risk populations [18,19] but not in community settings. Therefore, studies are needed to demonstrate the utility of the Microlife WatchBP Home A in opportunistic screening programs for AF in the community.

In Australia, opportunistic screening studies for AF using ECG were conducted in pharmacies [20] and general medical practices [21]. However, information on community-based opportunistic screening for AF is lacking, especially in the state of Tasmania, where the burden of stroke, hypertension, and AF-related hospitalisation is high [22]. Therefore, we developed and implemented a community-based opportunistic screening program to promote AF awareness and detection in Tasmania. We further investigated the performance of the Microlife WatchBP Home A for AF screening at public events.

## 2. Methods

### 2.1. Study Settings

The study, tagged “*What’s your beat*?”, incorporated state-wide voluntary screening for AF, designed along with an educational intervention for improving AF knowledge. The community-based opportunistic screening for AF was conducted at community venues, clubs, health expos, public events, and shopping centres in different demographic regions in Tasmania between March 2018 and May 2019.

### 2.2. Study Population

People aged ≥ 65 years were eligible to participate in the screening, except for those with a history of diagnosed AF, severe dementia, or cardiac arrhythmia or those having a pacemaker. The sample size was determined using a single proportion formula [23], adopting 1.5% as the expected prevalence of previously undiagnosed AF, based on the literature [20]. Assuming a 0.05 significance (alpha) level, a precision of 0.075 (half of the prevalence when the value is less than 10% [23]), and a z-value of 1.96 for a 95% confidence interval (CI), a minimum sample size of 999 participants was estimated.

### 2.3. Recruitment of Participants

Participants were recruited into the research through a community involvement approach [24], based on the Statement on the Consumer and Community Involvement in Health and Medical Research of the National Health and Medical Research Council [24]. The community was involved through collaboration with organisations such as the Council on the Ageing Tasmania (COTA), Hobart City Council (Positive Ageing Program), and Stroke Foundation Australia. Subsequently, these organisations facilitated recruiting the target population through invitations and sharing and displaying screening advertisements in their offices and social media pages. In addition, some organisations provided free transportation of participants to the screening venues.

The target population was also recruited from the community through a media campaign via magazines, newspapers, local radio stations, and announcements at screening venues and the distribution of flyers and posters at pharmacies and in public places.

### 2.4. Procedure

The screening for AF was performed using an automatic oscillometric BP monitor Microlife WatchBP Home A, Microlife AG Swiss Corporation, Switzerland [16]. The device had been validated in clinical settings with a reported sensitivity of 96.8% and a specificity of 88.8% [25]. The positive predictive value was calculated by dividing the number of participants with true-positive results (as determined following clinical review mentioned below) by the sum of true-positive and false-positive results [26].

Screening sessions were conducted by the primary investigator, research assistants, and at some points, nurses and a postdoctoral fellow. These individuals were trained on how to perform the AF screening, particularly using the screening device. Screening sessions were conducted based on a standard operating procedure (SOP); see Appendix A. Participants were informed verbally and in writing about consent to participate in the screening. Those who agreed to participate in the study were asked to sign a consent form and complete a data collection form. This form was used to obtain information such as age, sex, postcode (re-coded into the Socio-Economic Indexes for Areas (SEIFA)/Index of Relative Socio-Economic Advantage and Disadvantage (IRSAD) deciles, and the Accessibility/Remoteness Index of Australia plus (ARIA + 2016))*,* smoking status, presence of potential AF symptoms, and history of a medical condition (diabetes mellitus, heart failure, peripheral vascular disease, high blood pressure, heart attack, and stroke or transient ischaemic attack—TIA). Also recorded was participants’ awareness about AF, assessed using a question in the data collection form: “have you heard of the term atrial fibrillation?” (yes/no).

Subsequently, the blood pressure (BP) measurement was performed considering the recommendation of the device manufacturer and the Australian guideline for hypertension [16,27]. The BP of the participant was automatically measured using the Microlife WatchBP Home A, in triplicate, with the subject in a sitting position and having rested for five minutes. Participants were counselled appropriately based on the screening results, including an emphasis on the process not being diagnostic. Each participant with a positive AF screening result was provided with a referral letter to inform their general practitioner (GP) about the need for further review.

### 2.5. Follow-Up of Participants with Positive Screening Outcomes

The GP of each participant with a positive screening result was informed (via a telephone call) about the study, the result of the screening test for his/her patient, and the referral letter from the research team suggesting the need for clinical review (12-lead ECG or a 72 h Holster monitor) to confirm the presence of AF.

Subsequently, participants with positive screening results were followed up via a telephone call approximately 30 days after their screening. The participants were asked whether (i) they had visited the GP, (ii) the presence of AF had been confirmed or not by the GP, and (iii) AF management had been initiated. In addition, to further ensure the outcome of the visit to the GPs by the participants with positive screening results, letters in reply-paid envelopes were posted to the GPs. The GPs were asked to indicate the findings of any further testing regarding the potential AF in the referred patients and any AF management initiated.

### 2.6. AF Promotion at Screening Venues

AF promotional campaigns were delivered to the public through a ten-minute presentation about AF at some screening venues, posters, and the distribution of AF resource materials such as Australian Stroke Foundation flyers. AF education was also promoted through participants’ education via the teach-back technique. This technique was applied based on the recommendation of the Tasmanian Department of Health to check the understanding of the information provided [28].

### 2.7. Outcome Measures

The primary outcome was the prevalence of previously undiagnosed AF. The overall prevalence was calculated by dividing the number of participants with previously undiagnosed AF by the total sample size.

The stroke risk score of each participant was calculated using the CHA_2_DS_2_-VA score, as recommended by the Australian guideline for AF [8]. According to this scoring, the presence of congestive heart failure was assigned 1 point; high BP 1 point; age ≥ 75 years 2 points; diabetes mellitus 1 point; prior stroke/TIA 2 points; vascular disease 1 point; and age 65 to 74 years 1 point. Those with AF and a CHA_2_DS_2_-VA score of ≥2 were considered eligible for OAC therapy [8].

### 2.8. Statistical Analysis

Statistical analyses were conducted and reported based on Statistical Analyses and Methods in the Published Literature (SAMPL) guidelines for basic statistical reporting [29]. Statistical analyses were performed using the IBM SPSS Statistics for Windows, Version 25.0 (Armonk, NY, USA: IBM Corp.). Numerical data were checked for normality distribution using the Kolmogorov–Smirnov test and visual inspection of a histogram and Q-Q plot. Results of the descriptive analysis were presented as frequency (percentage) for categorical variables, and mean (SD) or median interquartile range (IQR) for numerical variables was reported. Chi-Square (or Fisher’s exact when expected frequency counts were less than five) tests were used to analyse the difference in proportion between two or more categorical variables.

## 3. Results

Seventy-nine screening sessions were conducted across Tasmania over the study period. Except for Flinders and King Islands, screening programs were performed in all the Tasmania regions, namely, Hobart and South region, Launceston and North region, North-West region, West Coast, and East Coast (Table 1).

### 3.1. Participants

A total of 1704 eligible participants were screened over the 12 months. The characteristics of the participants are summarised in Table 2. The median (IQR) age of the participants was 71.0 years (68.0 to 76.0). The oldest participants were 92 years old. A high proportion of participants was observed among the age category of 70 to 79 years (45.9%), females (59.0%), those living in inner regional areas (63.0%), and non-smokers (71.8%). The median (IQR) SEIFA-IRSAD decile was 7 (4.0 to 9.0).

A history of hypertension was present in 775 (45.5%) and diabetes mellitus (type 2) in 199 (11.7%) participants. Of the 1704 eligible participants, 259 (15.2%) had a systolic BP ≥ 160 mmHg at the time of screening, and all these were referred to their GP for further review. When participants were asked if they had heard about AF, 842 (49.4%) responded with yes and 862 (50.6%) with no.

### 3.2. Prevalence of Previously Undiagnosed AF

Of the total, 1654 (97.1%) had negative AF screening results, while possible AF was detected in 50 (2.9%) participants. Among those with positive screening results, one declined a follow-up, and another participant was lost to follow-up (due to incorrect contact details). Consequently, the other 48 (2.8%) participants with positive screening results visited their GP for further review. The mean (SD) time taken from the time of the screening until the GP visit was approximately 12 (9.5) days. In one of the participants, the presence of AF was not assessed based on advice from a GP following a discussion with the participant’s daughter (no further details were provided by the GP).

**Figure 1:**
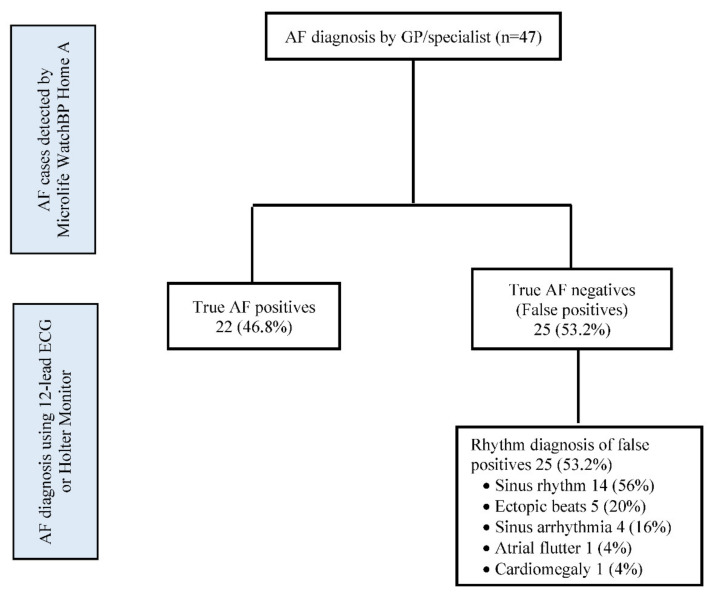
Diagnostic performance of Microlife WatchBP Home A (Positive predictive value of Microlife WatchBP Home A; 22/47 = 46.8%).

Of the 47 participants reviewed by their GPs to confirm the presence of AF, the Microlife WatchBP Home A correctly detected AF in 22 (46.8%) and produced 25 (53.2%) false-positive results (Figure 1). The positive predictive value of Microlife WatchBP Home A to identify AF was 46.8% (95% CI, 33.3 to 60.7). Among those with an AF-positive diagnosis, a follow-up with the GP confirmed a history of AF (not known by the participants) in 6 (27.3%), while 16 (72.7%) were found to have previously undiagnosed AF. Of the 25 participants with false-positive results, 14 (56%) were in sinus rhythm, 1 (4%) had cardiomegaly, and non-AF arrhythmias were identified in 10 (40%). Among those with non-AF arrhythmias, 4 (36.4%) had sinus arrhythmia, 5 (45.5%) had ectopic beats, and 1 (9.1%) had atrial flutter.

The overall prevalence of previously undiagnosed AF among the total participants was 0.9% (95% CI, 0.58 to 1.52). There was an increasing prevalence with age categories; for instance, from 0.3% (95% CI, 0.08 to 1.09) among the age group 65 to 69 years to 3.1% (95% CI, 1.57 to 5.98) in those ≥80 years. Compared with the with negative AF screening results, participants with previously undiagnosed AF were older (Mann–Whitney test (U) = 7330, *p* = 0.002; Table 3). There was a statistically higher proportion of participants with a history of peripheral vascular disease (chi-square test *χ*^2^ = 1.027, *p* = 0.001), any medical condition (*χ*^2^ = 9.003, *p* = 0.008), and potential AF symptoms (*χ*^2^ = 188.607, *p <* 0.001) among the subjects with previously undiagnosed AF, than among those with negative screening outcomes. Twelve (75%) (95% CI, 50.05 to 89.82) of the participants with previously undiagnosed AF reported a history of possible AF symptoms. The most common symptom was general fatigue, reported by seven (58.3%) of these participants.

### 3.3. Stroke Risk and Initiation of OAC Therapy

The mean (SD) CHA_2_DS_2_VA score of participants with previously undiagnosed AF was 2.8 (1.5). Fourteen (87.5%) of the participants had a score ≥2, of which 8 (57.1%) were males and 6 (42.9%) females. OAC was initiated in 12 (85.7%) of the 14 participants with CHA_2_DS_2_VA scores of ≥2 (eligibility for OAC therapy). Five of the six subjects with a history of AF (not known by the participants) had a CHA_2_DS_2_VA score of ≥2 and were thus eligible for OAC therapy. However, follow-up with the GP indicated that only one of them was already on OAC therapy.

## 4. Discussion

This is the first report of community-based opportunistic screening for AF in Australia using the Microlife WatchBP Home A. In this cohort of participants aged ≥ 65 years, a single time-point screening identified previously undiagnosed AF in 16 of 1704 (0.9%) participants. Following the screening program, OAC therapy was initiated in 12 participants. Additionally, 51% of participants became aware of AF through participation in the program, and the screening program was well embraced by the community. Given the low positive predictive value of the device recorded in this study (46.8% (95% CI, 33.3 to 60.7)), the use of the Microlife WatchBP Home A in community-based screening programs is unlikely to be cost-effective.

The prevalence of previously undiagnosed AF identified in this study was similar to previous studies on community-based opportunistic screening for AF using ECG [30,31]. However, the prevalence was higher than what has been reported in other community-based screening studies conducted with younger cohorts—in Germany among individuals aged 35 to 74 years old (0.5%) [32] and in the US among subjects aged ≥ 45 years (0.58%) [33]. Our finding is lower than the 1.4% pooled prevalence reported in a systematic review [34] and the 1.5% at community pharmacies in Australia [20]. These variations may be explained by the heterogeneity of detection methods, age groups, settings, and the target population.

The prevalence of previously undiagnosed AF reported in this study increased with age and was more frequently observed among males and those with additional AF risk factors. These results are consistent with the established epidemiological data on AF [35], a systematic review of screening studies for AF [34], and an opportunistic screening study conducted in Australia [20].

Although the NICE recommendation for the Microlife WatchBP Home A was based on its diagnostic performance among high-risk patients in clinical settings, its positive predictive value in this study was considered low (46.8%). This value was lower than the 68% obtained in validation studies among high-risk cardiology outpatients [36] and the 80% using other devices such as the iECG (AliveCor Kardia app) [37]. However, our positive predictive value was comparable with the values observed when the device was used in validation studies among high-risk patients in primary care settings (42.4% and 44%) [18,19]. It is possible that the performance of the device in this study was affected by the relatively noisy nature of the screening venues (community-based centres), where artefacts are possible [16]. Another reason could be the high number of participants (40%) without additional risk factors (underlying medical conditions) in our study. The literature suggests that the diagnostic performance of a screening device is more likely to be higher in those with additional risk factors [38].

The high rate of false-positive results has been widely reported as one of the major drawbacks of AF detection using BP monitors [36], including the Microlife WatchBP Home A [18,19]. Some experts have opined that a high rate of false positives produced by a device may lower the yield and cost-effectiveness of a screening program [39]. This limitation suggests the need to improve the diagnostic performance of screening devices [13].

The community-based screening for AF using the BP monitor was well received by participants and collaborators in Tasmania. Other studies also reported a high degree of acceptability of opportunistic screening for AF to the target population in Sydney, Australia [40].

### Strengths and Limitations

This study has several strengths. First, the screening program was free. Removing economic barriers may have increased the uptake of screening. Second, participants were recruited from remote, rural, and regional urban areas across Tasmania. Third, while some studies relied primarily on AF case detection without confirmation using ECG [41], all participants with positive screening results in our study were linked up with a referral pathway for further medical care.

The limitations included the use of a single time point for screening, reliance on self-reporting, and the performance of the device used in the study settings. It is likely that this method may miss many participants with paroxysmal AF, who may not be in AF during screening. Conversely, patients may have been in paroxysmal AF at the time of screening with the Microlife WatchBP Home A but not when subsequently investigated by their doctor. There was reliance on self-reporting to determine other medical conditions and the presence of possible AF symptoms. The high rate of false-positive results produced by the Microlife WatchBP Home A may be explained, in part, by the sensitive nature of the device to noisy environments. Future studies should consider this inherent drawback.

## 5. Conclusions

Community-based opportunistic screening for AF using the Microlife WatchBP Home A identified 0.9% of older people with previously undiagnosed AF in Tasmania. The device had a positive predictive value of 46.8%. Thus, given its low performance in this setting, its application in community-based opportunistic screening for AF is unlikely to be cost-effective.

## Figures and Tables

**Table 1:** Locations of screening sessions.

Tasmania Regions	Regional Areas	No. of Screening Sessions	Nature of Venue
Hobart and Southern Tasmania	Bruny Island	1	Community hall
	Greater Hobart	50	Community hall, festival, shopping centre, street
	Huonville	3	Community hall, festival
	Cygnet	1	Community hall
North West Tasmania	Devonport	1	Festival
	Burnie	1	Community hall
	Sheffield	1	Community hall
	Ulverstone	1	Community hall
	Port Sorell	1	Community hall
West Coast Tasmania	Queenstown	1	Festival
Launceston and Northern Tasmania	Launceston	11	Community hall, market, festival
	Deloraine	1	Community hall
	Carrick	1	Community hall
	Evandale	1	Festival
	Bridport	1	Community hall
East Coast Tasmania	St Helens	1	Community hall
	Bicheno	1	Community hall
	Fingal	1	Community hall
**Total**		**79**	

**Table 2:** Characteristics of the study participants (*n* = 1704).

Variables		Median (IQR)	Frequency (%)
Age		71.0 (68.0 to 76.0)	
Age categories (year)	65 to 69		663 (38.9)
	70 to 79		782 (45.9)
	≥80		259 (15.2)
Sex	Female		1006 (59.0)
	Male		698 (41.0)
Socioeconomic status	Deciles	7.0 (4.0 to 9.0)	
Remoteness	Inner regional		1074 (63.0)
	Outer regional		528 (31.0)
	Remote/very remote		102 (6.0)
Smoking status	Non-smoker		1224 (71.8)
	Ex-smoker		422 (24.8)
	Current smoker		58 (3.4)
History of a medical condition	Diabetes mellitus 1		16 (0.9)
	Diabetes mellitus 2		199 (11.7)
	Heart failure		39 (2.3)
	PVD		60 (3.5)
	High blood pressure		775 (45.5)
	Heart attack		110 (6.5)
	Stroke/TIA		102 (6.0)
	Other medical conditions		291 (19.1)
	None		659 (38.7)
Systolic BP at screening (mmHg)	<20		174 (10.2)
	120 to 129		271 (15.9)
	130 to 139		403 (23.7)
	140 to 159		597 (35.0)
	≥160		259 (15.2)
AF Awareness	Yes		842 (49.4)
	No		862 (50.6)

TIA, Transient Ischaemic Attack; PVD, Peripheral Vascular Disease; IQR, Interquartile Range.

**Table 3:** Comparison of participants with previously undiagnosed AF and those with negative screening results.

Variables		Previously Undiagnosed AF *n* = 16, *n* (%)	Negative Screening Outcomes *n* = 1679, *n* (%)	*p*
Median (IQR) deciles	SEIFA-IRSAD	7.5 (4.25 to 8.75)	7.0 (4.0 to 9.0)	0.652 ^a^
Median (IQR) age (years)		79 (70.6 to 85.3)	71.0 (68.0 to 76.0)	**0.002 ^a^**
Age categories (years)	65 to 69	2 (12.5)	657 (39.1)	**<0.001 ^b^**
	70 to 79	6 (37.5)	773 (46.0)	
	80 and above	8 (50.0)	249 (14.8)	
Sex	Female	9 (56.3)	992 (59.1)	0.819 ^b^
	Male	7 (43.8)	687 (40.9)	
Location	Inner regional	12 (75.0)	1056 (62.9)	0.563 ^b^
	Outer regional	3 (18.3)	623 (37.1)	
	Remote/very remote	1 (6.3)	101 (6.0)	
Smoking status	Non-smoker	9 (56.3)	1209 (72.0)	0.367 ^b^
	Ex-smoker	6 (37.5)	414 (24.7)	
	Current smoker	1 (6.3)	56 (3.3)	
Medical condition	Yes	15 (93.8)	1030 (61.0)	**0.007 ^b^**
	No	1 (6.3)	658 (39.0)	
	Diabetes mellitus 1	1 (6.3)	15 (0.9)	0.141 ^c^
	Diabetes mellitus 2	4 (25.0)	192 (11.4)	0.091 ^b^
	Heart failure	0 (0.0)	38 (2.3)	-
	PVD	3 (18.8)	55 (3.3)	**0.001 ^c^**
	High blood pressure	9 (56.3)	762 (45.4)	0.385 ^b^
	Heart attack	2 (12.5)	106 (6.3)	0.313 ^b^
	Stroke/TIA	1 (6.3)	99 (5.9)	0.952 ^b^
	None	1 (6.3)	658 (39.0)	**0.008 ^b^**
Systolic BP at screening (mmHg)	<120	1 (6.3)	172 (10.2)	0.110 ^b^
	120 to 129	2 (12.5)	269 (16.0)	
	130 to 139	1 (6.3)	399 (23.8)	
	140 to 159	8 (50.0)	585 (34.8)	
	≥160	4 (25.0)	251 (15.1)	
History of symptoms	Yes	12 (75.0)	58 (3.5)	**<0.001 ^b^**
	No	4 (25.0)	1621 (96.5)	
AF Awareness	Yes	8 (50.0)	829 (49.4)	0.960 ^b^
	No	8 (50.0)	850 (50.6)	

^a^ Mann–Whitney test; ^b^ Chi-Square test; ^c^ Fisher’s Exact test; PVD, Peripheral Vascular Disease; TIA, Transient Ischaemic Attack; AF, Atrial Fibrillation; Significant results (*p*-value < 0.05) presented in bold.

## Data Availability

The data presented in this study are available on reasonable request from the corresponding author.

## Appendix A

Standard operating procedure for conducting screening for AF.
